# Cross-sectional survey of asymptomatic malaria in Dak Nong province in the Central Highlands of Vietnam for the malaria elimination roadmap

**DOI:** 10.1371/journal.pone.0258580

**Published:** 2021-10-20

**Authors:** Huynh Hong Quang, Marina Chavchich, Nguyen Thi Minh Trinh, Nguyen Duc Manh, Michael D. Edstein, Nicholas J. Martin, Kimberly A. Edgel

**Affiliations:** 1 Institute of Malariology, Parasitology and Entomology, Quy Nhon, Vietnam; 2 Australian Defence Force Malaria and Infectious Disease Institute, Brisbane, Australia; 3 Military Institute of Preventive Medicine, Hanoi, Vietnam; 4 U.S. Naval Medical Research Unit TWO, Singapore, Singapore; Instituto Rene Rachou, BRAZIL

## Abstract

Asymptomatic parasite carriers represent a “silent” infective reservoir for malaria transmission and contributes to malaria persistence. However, limited data are available on asymptomatic malaria in Vietnam. Between November 2018 and March 2019, we conducted a malaria epidemiological survey of asymptomatic people (children ≥ 10 years old and adults ≥18 years old, n = 2,809) residing in three communes in Tuy Duc district, Dak Nong province in the Central Highlands of Vietnam. Based on the national stratification of malaria risk, Dak Buk So, Dak Ngo and Quang Truc communes were classified by the National Malaria Control Programme as low, moderate and high malaria endemic areas, respectively. Using participants’ finger prick blood samples, malaria parasites were detected by one-step reverse transcription-quantitative polymerase chain reaction (RT-qPCR). The median age (Interquartile Range) for adults and children were 35 years (26–50) and 12 years (11–14), respectively. The prevalence of asymptomatic malaria was 1.7% (22/1,328), 3.5% (31/890) and 12.2% (72/591) for participants from Dak Buk So, Dak Ngo and Quang Truc, respectively. The prevalence of asymptomatic malaria was lower in children compared to adults: 2.6% (9/352) versus 4.7% (116/2,457) (Odds Ratio 0.53, 95% Confidence Interval 0.28 to1.02). Ownership of long-lasting insecticide-treated bed nets and hammocks was 97.1%, 99.0% and 94.7% for participants in Dak Buk So, Dak Ngo and Quang Truc, respectively, however, only 66.0%, 57.3% and 42.8% of the participants reported using bed nets every night. Of the several risk factors examined, going to the forest two weeks prior to enrolment into the study and sleeping in the forest had a significant association with participants being infected with asymptomatic malaria in Quang Truc, but not in the other two communes. Knowledge of the prevalence and distribution of asymptomatic malaria will help design and evaluate future intervention strategies for malaria elimination in Vietnam.

## Introduction

Nations within the Greater Mekong Sub-region (GMS), including Vietnam, have adopted the goal to eliminate malaria by 2030 [1]. In Vietnam, the morbidity and mortality of malaria have sharply declined over the past 8 years from 17,229 malaria cases in 2010 to 4,813 cases in 2018, and malaria deaths fell from 21 in 2010 to 1 in 2018 [2]. This marked decline in malaria cases provides the opportunity for Vietnam to pursue malaria elimination by 2030. In order to use targeted approaches for malaria elimination, the country has been stratified at the communal level into fives zones according to malaria risk, with zones 1 and 2 declared free of malaria, whereas zones 3, 4 and 5 are low, moderate and high malaria endemic areas, respectively (WHO, 2018). According to the National Strategy for Malaria Control and Elimination, the immediate goal is to eliminate malaria in low endemic areas, whereas in moderate and high endemic areas the priority is to actively control malaria. However, a critical challenge for malaria elimination is asymptomatic carriers with mostly submicroscopic parasitemia, who provide an infective “silent” reservoir for malaria transmission and contribute to the persistence of infections [3–12].

The global prevalence of asymptomatic malaria varies widely from 0.4% to 90.6% in different endemic settings with the median parasite density correlating with the prevalence of asymptomatic infections and ranging from 1 to 1,336 parasites per μL [13]. A recent study in Western Kenya, in an area of high malaria transmission, revealed that asymptomatic infections were responsible for 94.6% of malaria transmission [9]. Even though in low transmission areas asymptomatic low-density parasite infections are associated with lower gametocyte densities [14], they still contribute to malaria transmission [10, 13, 15, 16]. Studies in Vietnam and Lao-PDR have provided evidence of persisting and oscillating submicroscopic malaria infections that frequently led to high-density infections, thus sustaining malaria transmission [7, 8]. Combination of antimalarial treatments with preventive measures, such as long-lasting insecticide-treated nets (LLINs), in an area of high transmission in Senegal, resulted in the prevalence of asymptomatic malaria declining from 36% in 1993 to 1% in 2013, with a parallel decrease in the median pyrogenic parasitemia threshold from 2,500 parasite/μL to 100 parasite/μL [17]. Asymptomatic infections are also responsible for sustaining the malaria infectious reservoir during the dry season, when the mosquito vectors are absent [18, 19].

In the absence of a curative drug treatment, asymptomatic *Plasmodium falciparum* and *P*. *vivax* infections persist frequently for months or years [7, 20, 21], posing many additional risks, including anemia, pregnancy complications, systemic bacterial infections, and impairment of cognitive functions in children (reviewed in [22]). Anemia, associated with asymptomatic malaria infections is positively correlated with the parasite density and frequency of recurrent symptomatic infections. In areas where asymptomatic malaria is common, it is difficult to prove its association with anemia because of the presence of other conditions that may also cause anemia [22]. Targeting of asymptomatic malaria with effective measures, such as drug treatment and LLINs should accelerate malaria elimination.

Gaining an understanding of the prevalence and distribution of asymptomatic malaria in Vietnam will help design intervention strategies to achieve malaria elimination. Surveys of submicroscopic malaria infections, detectable only by polymerase chain reaction (PCR) have revealed asymptomatic malaria to be common near and in forested areas of south-central [7, 23], central [23–25] and south-western Vietnam [7, 26]. Retrospective analysis of filter paper blood spots collected from participants in a malariometric survey in 2004 in a rural area of Ninh Thuan province in south-central Vietnam revealed, that of 671 PCR positive samples, only 331 (49.3%) subjects were detected by blood film microscopy and thus indicating a high percentage of submicroscopic malaria [25]. In 2009, in a survey of 1,450 participants living in a remote forested valley of Quang Nam province in Central Vietnam the malaria prevalence was 7.8% (113/1,450) by blood film microscopy and 22.6% (74/327) by PCR in randomly collected blood samples [23], suggesting a high percentage of asymptomatic malaria infections in the local population. In 2013, using an ultra-sensitive DNA-based PCR (uPCR) method, with a lower limit of detection of 22 parasites/mL of blood, that requires a high volume of venous blood (up to 3 mL) [27], the prevalence rate of submicroscopic malaria was 12.0% (239/1,992) in Vietnamese people from two villages in south-western Binh Phuoc province and in two villages in Ninh Thuan province [26]. The asymptomatic *Plasmodium* spp. prevalence rate for the four villages ranged from 7.4% to 14.7%. The uPCR method was also able to determine that of the asymptomatic participants, 2.3% (45/1,992), 4.0% (79/1,992), 1.6% (31/1,992) and 4.2% (84/1,992) were infected with *P*. *falciparum*, *P*. *vivax*, mixed *P*. *falciparum* and *P*. *vivax* and other than *P*. *falciparum* and *P*. *vivax* malaria, respectively [26].

In other Southeast Asian countries where uPCR has been used, high asymptomatic malaria prevalence rates were detected in three western Cambodian villages (15.8%; 229/1,447) and in four villages on the Thailand-Myanmar border (33.9%; 520/1,536) [26]. In 2015, in 18 Lao-PDR villages in Southern Savannakhet province, uPCR detected submicroscopic *Plasmodium* infections in 19.7% (175/888) of the study population [8]. These findings revealed that asymptomatic and mostly submicroscopic malaria is common in Southeast Asian countries, including Vietnam and can reach high prevalence rates.

Other than the studies conducted in participants from Binh Phuoc, Quang Nam and Ninh Thuan provinces [7, 23, 25, 26], there is no asymptomatic malaria prevalence data for provinces in the Central Highlands of Vietnam, particularly in areas of artemisinin and piperaquine resistance [28, 29]. The aim of this study was to determine the prevalence of asymptomatic malaria in people residing in three communes in Tuy Duc district, Dak Nong province in the Central Highlands of Vietnam using a highly sensitive one-step reverse transcription-quantitative polymerase chain reaction (RT-qPCR) assay [30] on finger prick blood samples. Factors, such as age, gender, body temperature, hemoglobin (Hb) concentration, occupation, going to the forest and use of LLINs and their association with asymptomatic malaria were analysed. The findings from this study will assist the National Malaria Control Programme (NMCP) in decision making and developing malaria elimination strategies for Vietnam.

## Materials and methods

### Study area and study design

**Fig 1:**
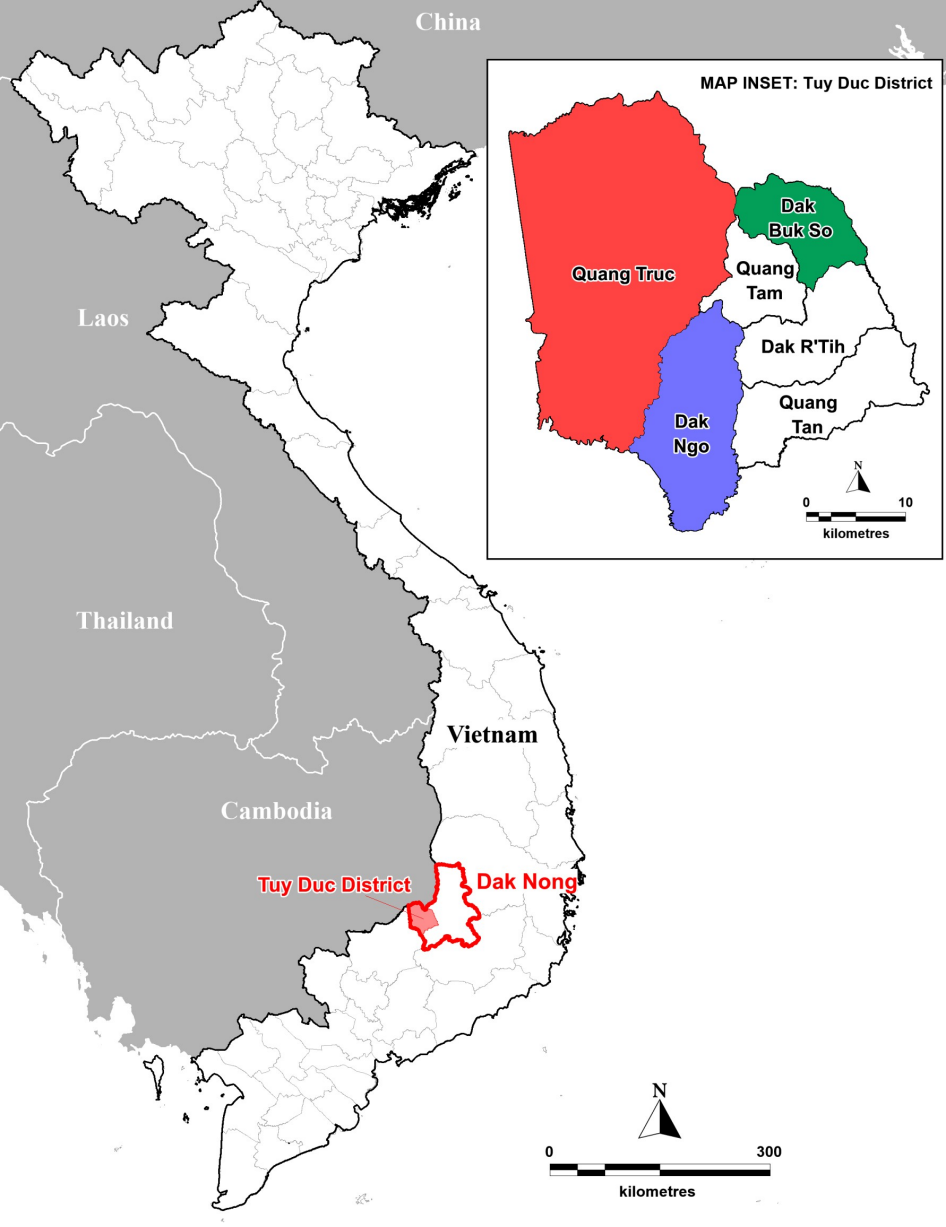
Study sites of three communes in Tuy Duc district, Dak Nong province, Vietnam. Custom map produced using MapInfo Professional v15.0.2 (Pitney Bowes Software Inc. 2015, Stamford, CT; https://www.pitneybowes.com) Geographic Information System (GIS) mapping software.

Tuy Duc district, Dak Nong province borders with Cambodia and Binh Phuoc province in south-western Vietnam (**Fig 1**). The district has 17 ethnic minority groups: the two major ethnic groups are the M’Nong and H’Mong people. The main income for the people living in the district is generated from agricultural production, including rice farming near their homes or in forested areas, as well as from coffee, pepper and potato plantations.

The cross-sectional survey was carried out in people from three communes, Dak Buk So, Dak Ngo and Quang Truc, which were stratified by NMCP as low, moderate and high malaria risk areas, respectively [31]. The study was carried out over two periods and was divided into two cohorts, with approximately the same number of participants recruited for each cohort. The 1^st^ and 2^nd^ cohorts of asymptomatic people (i.e., subjects without symptoms of malaria such as fever, headache, nausea and loss of appetite) were surveyed at the end of the wet season (November-December 2018) and during the dry season (February-March 2019), respectively. Individuals, who lived near the communal health stations and preferably worked in rural areas, were invited to participate in the study and different participants were recruited for each cohort.

### Participants and questionnaire

The inclusion criteria for participants were: absence of malaria symptoms, age ≥10 years and willingness to provide a finger prick capillary blood sample. Participants with tympanic temperatures <38.0°C were considered to be afebrile. People who were recruited into the study were invited to complete a questionnaire consisting of questions of demographic characteristics, recent medical history, travel in the past two weeks and malaria prevention measures, such as sleeping under a bed net and using a topical repellent.

### Sample size

Based on the asymptomatic prevalence rates seen in Vietnamese villages in Binh Phuoc and Ninh Thuan provinces [26], it was expected that areas of low, moderate and high malaria risk would have asymptomatic prevalence rates of 5%, 10% or 15%, respectively. Using the formulae N = Z^2^ p (1-p) /d^2^, where N is the sample size, Z- statistics equals 1.96 at a confidence level of 95% and applying p (prevalence) values of 0.05, 0.10 and 0.15 with respective d (precision) values of 0.012, 0.02 and 0.03, the minimum numbers of people to be recruited were 1,267 in Dak Buk So, 864 in Dak Ngo and 544 in Quang Truc. These numbers were increased by 5% for unexpected withdrawals with a total of 2,809 participants to be recruited.

### Data and sample collection

Participating adults and children were examined by a study doctor. Their vital signs (i.e., pulse and respiratory rates) and clinical information, such as body weight, body temperature and recent medical history were collected. A finger prick capillary blood sample (~130 μL) was collected from each participant and transferred to a Becton-Dickinson (BD) microtube (500 μL) containing EDTA as an anticoagulant and gently mixed. Fifty-μL was transferred to an Eppendorf^®^ LoBind microcentrifuge tube containing 250 μL of RNAprotect reagent (Qiagen GmbH, Germany) and stored at -20°C until shipment on dry ice to the Institute of Malariology, Parasitology and Entomology, Quy Nhon (IMPE-QN) for laboratory analysis.

### Detection of malaria infections and measurement of blood hemoglobin (Hb) concentrations

Malaria RDT (SD Bioline Malaria Ag Pf/Pan, Kyonggi, Republic of Korea) is based on the detection of histidine-rich protein 2 (HRP-2) produced by *P*. *falciparum* and lactate dehydrogenase (pLDH) of the four *Plasmodium* species; *P*. *falciparum*, *P*. *vivax*, *P*. *malariae* and *P*. *ovale*. Testing was carried out according to the manufacturer’s instructions. Thick and thin blood films (quantity two) were prepared for malaria diagnosis. The dried thin films were fixed with methanol. The blood films were stained with 4% Giemsa for 45 min and examined at a magnification of 1000× by two trained independent microscopists. Parasites were counted per 200 white blood cells (WBCs) and parasite density was calculated by assuming a WBC count of 8,000/μL in accordance with the WHO guidelines [32]. A blood film was considered negative when examination for 1,000 WBC revealed no asexual or sexual parasites.

To determine the participant’s Hb concentration, 7 μL of mixed EDTA blood from the BD microtube was transferred to a CareSTART Hemoglobin Monitoring System (Access Bio, New Jersey, USA) and the value was recorded.

### Detection and *Plasmodium* speciation by RT-qPCR

Blood samples preserved in RNAprotect reagent were used for RNA/DNA extraction using an RNeasy kit (Qiagen GmbH, Germany). The RNA/DNA extraction procedure was tested and modified to accommodate the extraction using 300 μL sample volume as follows: 1.1 mL of RLT-β buffer containing β-mercaptoethanol was added to the 300 μL sample followed by the addition of 1.4 mL of 70% ethanol. The procedure was then conducted in accordance with manufacturer’s instructions with no DNA elimination step carried out. Parasite RNA and DNA were eluted in 50 μL RNAase-free water (Qiagen GmbH, Germany).

Malaria positive samples were determined in a one-step RT-qPCR analysis, targeting 18S small sub-unit ribosomal RNA (SSU rRNA) gene transcripts for a simultaneous detection of the four *Plasmodium* species (*P*. *falciparum*, *P*. *vivax*, *P*. *malariae* and *P*. *ovale*) using primers QMAL_Fw- TTAGATTGCTTCCTTCAGT(A/G)CCTTATG and QMAL_Rev-GTTGAGTCAAATTAAGCCGCAA as previously described [30], with the exception of using Sybr Green master mix (Qiantitect Sybr Green one-step RT-qPCR kit Qiagen GmbH, Germany, ref. 204243) instead of Taqman probes to improve sensitivity. Briefly, the RT-qPCR reactions were set in 12 μL and contained 800 nM of QMAL primers, 0.3 μL of RT-mix and 2–4 μL of RNA/DNA template. The one-step RT-qPCRs were carried out in a AB7300 (Thermo Fisher Scientific Inc., USA) machine with cycling conditions as follows: 50°C for 30 min, 95°C for 15 min, followed by 45 cycles of 94°C for 15 sec and 58°C for 1 min. For species determination either Pfal_Fw-TATTGCTTTTGAGAG GTTTTGTTACTTTG and Pfal_Rev-TATTCCATGCTGTAGTATTCAAACACAA for *P*. *falciparum* or Pv-Fw- GCTTTG TAATTGGAATGATGGGAA T and Pv_Rev- ATGCGCACAAAGTCGATACGAAG for *P*. *vivax* were used in respective RT-qPCR reactions as described above.

To test the sensitivity of the assay, standards were prepared from highly synchronous parasite cultures of the *P*. *falciparum* D6 laboratory line (originally from Sierra-Leone, Africa) containing predominantly young rings (>95%). Aliquots (50 μL) of cultured parasites at 5.5% parasitemia (quantified by microscopy) and 46% hematocrit in human plasma were prepared, to which RNAprotect reagent (250 μL) was added and the standards were stored at -80°C. Ten-fold serial dilutions with blood/RNAprotect mixture (up to 4 x 10^−9^ fold) were made to test the sensitivity of detection. The detection was reproducibly achieved with a threshold cycle (Ct) of 32 in at least 10 independent experiments, using 2 μL (out of 50 μL) of RNA/DNA template (in water) containing equivalent of 0.001012 parasites, corresponding to 0.0253 parasites/μL (or 25 parasites/mL) in a positive control dilution. For lower dilutions, the detection of positive samples became stochastic. It follows that if at least one parasite is present in the initial volume of finger prick capillary blood sample (50 μL) the assay would be sufficiently sensitive to detect positive samples by RT-qPCR. The efficiency and sensitivity of detection for the other three *Plasmodium* species was not tested.

Following detection of malaria positive samples, the *Plasmodium* speciation was carried out as described in Wampfler *et al*. [30] by species specific RT-qPCRs. For quality assurance, in addition to the participants’ samples, each RT-qPCR plate contained an RNA extraction efficiency control, qPCR positive and negative controls. The RNA efficiency controls were obtained by extracting RNA from a *P*. *falciparum* standard dilution containing approximately 250 parasites, with an expected Cts of 19–20. The RNA/DNA extracted from a *P*. *falciparum* standard dilution containing 25 parasites was aliquoted and used as a RT-qPCR positive control (Ct of 23–24). The positive samples were identified by analysis of the melting curve profiles of the RT-qPCR products and compared to the melting temperatures (Tm) of the RT-qPCR products and those obtained from the positive controls. The samples with 78°C <Tm<80°C were selected as possibly positive and subsequently confirmed as positive by at least one more RT-qPCR.

### Statistical analysis

Statistical analysis of the demographic and clinical data was performed using SigmaStat (version 3.0 Jandel Scientific, CA, USA). Descriptive statistics were used to summarize baseline values and demographic data. Variable comparisons were analysed using the Kruskal-Wallis one-way analysis of variance (ANOVA) on ranks and Mann-Whitney rank sum test. Values of normally distributed data were expressed as means with standard deviations (SD) and non-normally distributed data as medians with interquartile ranges (IQR). Comparison between blood Hb concentrations in RT-qPCR-positive and -negative participants was carried out using the Fisher’s exact test of contingency tables for Hb values of <11 g/dL and ≥11 g/dL using GraphPad Prizm Version 9.0.1 software package (GraphPad Prizm, Inc. State, USA). Risk analysis using contingency tables was also used to evaluate association of asymptomatic malaria with factors, such as occupation (farmers, forest rangers or students), time spent in the forest and use of LLINs and repellents. Multiple logistic regression analysis was used to analyse the association of physical characteristics (e.g., age, gender, body weight, body temperature, pulse, respiratory function) with RT-qPCR positivity (dependent variable) using GraphPad Prizm software. Differences were considered statistically significant when *P*<0.05.

### Ethics approval and consent to participate

Ethics approval for the study was obtained from the Institutional Review Board of the Institute of Malariology, Parasitology and Entomology Quy Nhon (Ref. No. 533/VSR-LSDT) and mutual recognition by the Australian Government Departments of Defence and Veterans’ Affairs Human Research Ethics Committee (Ref. No. 2018/R32250633). Extramural research review was also conducted in accordance with the U.S. Navy Human Research Protection Program (HRPO.NMRCA 2018.0012) in compliance with all applicable federal regulations governing the protection of human subjects. All adults gave written informed consent and children (≥10 to < 18 years old) gave written assent with permission from their parent or guardian to be in the study.

## Results

### Demographic and clinical characteristics of participants

The demographics of the 2,809 participants from the three communes are shown in **Table 1**. Of these participants, 37.9% (1,065/2,809) were males and 87.5% (2,457/2,809) were adults. The median (IQR) ages for adults and children were 35 years (26–50) and 12 years (11–14), respectively. All children were greater than 10 years old. The median (IQR) body weights were 53 kg (49–59) for adults and 39 kg (34–41) for children. The participants were afebrile with a median (IQR) tympanic body temperature of 36.9°C (36.7–37.0). Multiple comparisons of the physical characteristics of the participants revealed statistical differences (*P*<0.05) in the age and body weight of adults and children between the three communes, whereas there were no differences in gender and body temperature in adults and children.

**Table 1:** Characteristics of the study populations at Dak Buk So, Dak Ngo and Quang Truc communes, Tuy Duc district, Dak Nong province, Central Highlands of Vietnam.

	Dak Buk So	Dak Ngo	Quang Truc
No. of participants	1,328	890	591
Male % (Male/Female)	38.9% (516/812)	36.9% (328/562)	37.4% (221/370)
Adults (≥ 18 yrs) %	88.6% (1,176/1,328)	89.6% (797/890)	81.9% (484/591)
Children (≥ 10 to < 18 yrs) %	11.4% (152/1,328)	10.4% (93/890)	18.1% (107/591)
Adult (yrs)*	36 (27–51)	33 (25–47)	37 (26–50)
Child (yrs)*	12 (11–14)	12 (10–13)	13 (11–14)
Adults body weight (kg)*	54 (49- 60)	53 (49–59)	52 (49–58)
Child body weight (kg)*	40 (38–41)	33 (30–39)	39 (37–42)
Adult body temp (°C)	36.9 (36.7–37.0)	36.9 (36.7–37.1)	36.9 (36.8–37.1)
Child body temp (°C)	36.9 (36.7–37.0)	36.9 (36.6–36.9)	36.9 (36.8–37.0)

Values are median (interquartile range)

* denotes, where significant differences (*P*<0.05) were found by multiple comparisons between the three communes.

None of the 2,809 participants in the cross-sectional asymptomatic malaria prevalence survey reported malaria symptoms or had taken antimalarial drugs for 28 days prior to enrolment. All but one participant (QT554) were RDT negative and three were blood film microscopy positive for malaria infections: two participants from Quang Truc, QT528 (*P*. *falciparum*—RDT negative), QT554 (*P*. *vivax*—RDT positive) and one participant from Dak Buk So, DBS1026 (*P*. *falciparum*—RDT negative). Despite microscopically detected parasitemia, these three participants did not report any malaria symptoms and had body temperatures <37.5°C at enrolment, but two participants with *P*. *falciparum* infections developed malaria symptoms soon after and were treated for their malaria infections.

### Prevalence of asymptomatic malaria in study participants

**Fig 2:**
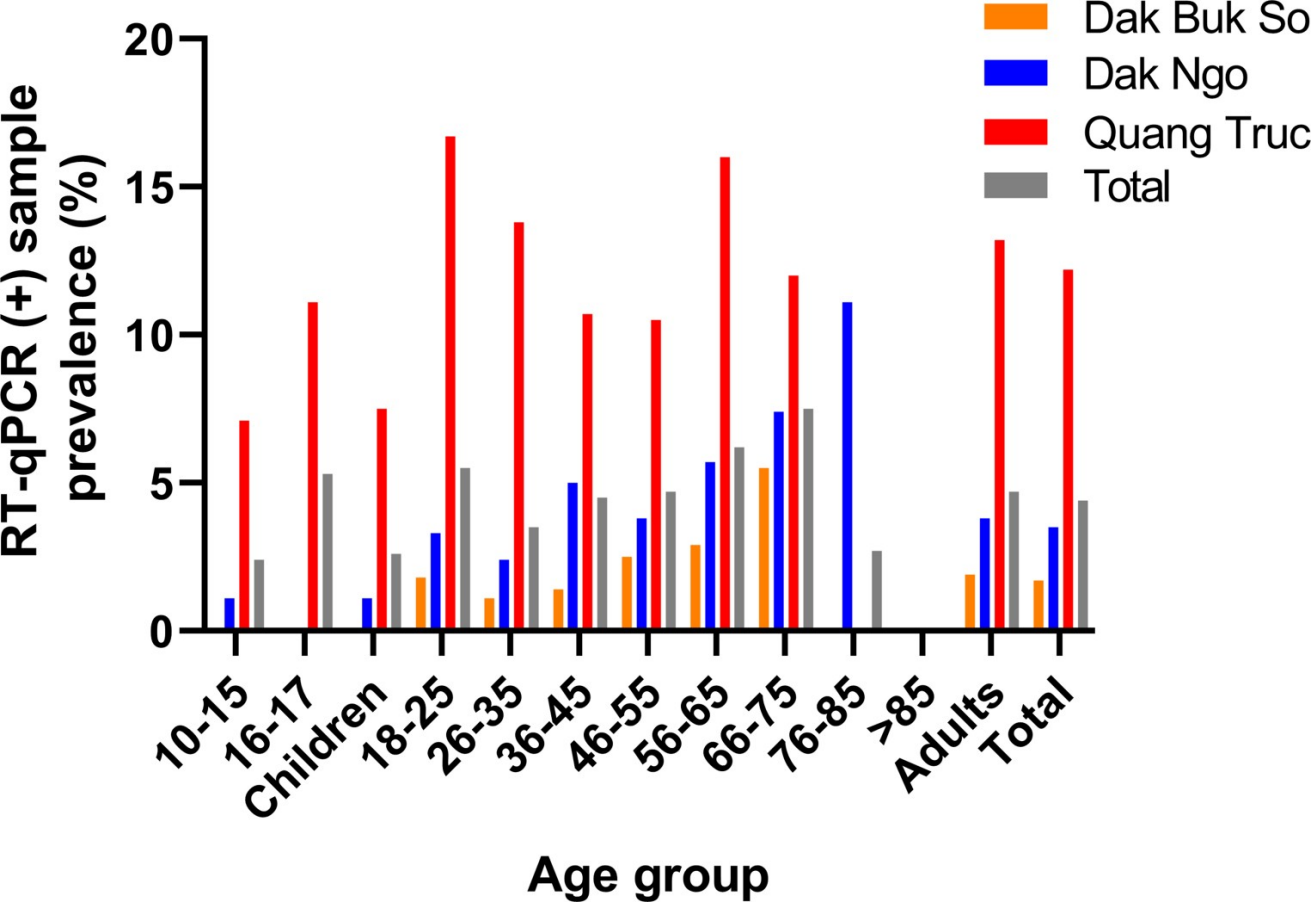
RT-qPCR positive sample (asymptomatic malaria) prevalence in participants of different age groups in Dak Buk So, Dak Ngo and Quang Truc communes, Tuy Duc district, Dak Nong province, Central Vietnam in 2018–2019.

The point prevalence of participants infected with asymptomatic malaria determined by RT-qPCR were 4.4% (62/1,413) for the 1^st^ cohort and 4.5% (63/1,396) for the 2^nd^ cohort, with an overall prevalence of 4.4% (125/2,809) (**Table 2**), with no statistical difference in prevalence between the two cohorts (*P>*0.05). The point prevalence of asymptomatic malaria determined by RT-qPCR were 1.7% (22/1,328), 3.5% (31/890) and 12.2% (72/591) for participants from Dak Buk So, Dak Ngo, and Quang Truc, respectively (**Table 2, Fig 2**). This prevalence of asymptomatic malaria was consistent with the stratification of malaria risk and the incidence of symptomatic malaria infections reported by the NMCP [33] for 2018, with Dak Buk So (low risk) having the lowest number of blood film positive malaria cases, Dak Ngo (moderate risk) and Quang Truc (high risk) having the highest number of acute malaria infections (**Table 2**).

**Table 2:** Asymptomatic malaria cases in Dak Buk So, Dak Ngo and Quang Truc communes by RT-qPCR (1^st^/ 2^nd^ cohorts) analysis.

Malaria risk ranking*	Commune	Analysed samples (n = 1^st^/ 2^nd^)	Negative samples		Positive samples		2018 Positive symptomatic**
			by RT-qPCR		by RT-qPCR		
		No.	(n = 1^st^/ 2^nd^)	%	(n = 1^st^/ 2^nd^)	%	%
			No.		No.		(n/N)
Low	Dak Buk So	(663/665)	(653/653)	98.3	(10/12)	1.7	0.1
		1,328	1,306		22		(1/883; 1 Pf)
Moderate	Dak Ngo	(445/445)	(429/430)	96.5	(16/15)	3.5	0.6
		890	859		31		(14/2,272; 8 Pf, 6 Pv)
High	Quang Truc	(305/286)	(269/250)	87.8	(36/36)	12.2	3.0
		591	519		72		(57/1,903; 29 Pf, 27 Pv, 1 Pf/Pv)
	**Total**	**2,809**	**2,683**	**95.6**	**125**	**4.4**	**1.4**
							**(72/5,058)**

*Malaria risk area stratification by the National Malaria Control Program [31].

**Based on passive case detection data for people presenting at the three commune health stations in 2018 with the number of patients diagnosed with *P*. *falciparum* (Pf), *P*. *vivax* (Pv) and mixed malaria infections by blood parasite confirmed microscopy per the number of people screened for malaria infection [33].

1^st^ cohort screened November-December 2018 (wet season); 2^nd^ cohort screened February-March 2019 (dry season).

Across the three communes, of the *Plasmodium* positive cases, only 7.2% (9/125) were detected in children (median age: 14 years, IQR 12–14) and 92.8% (116/125) in adults (median age: 37 years, IQR 25–49), of which 60.8% (76/125) were females. The overall prevalence of participants infected with asymptomatic malaria between the three communes was statistically different (*P*<0.05).

The prevalence of RT-qPCR positive samples amongst different age groups in the three communes is shown in **Fig 2 and S1 Table in S1 Appendix.** In children (≥10 to < 18 years old) there were no asymptomatic malaria cases detected in Dak Buk So (0/152) and only 1.1% (1/93) detected in Dak Ngo, whereas in Quang Truc 7.5% (8/107) of surveyed children were positive for asymptomatic malaria compared to an overall prevalence in adults of 13.2% (64/484, Relative Risk, RR = 0.57; Odds Ratio, OR = 0.53; 95% CI: 0.80 to 2.57, *P*>0.05) (**Fig 2, S1 Table in S1 Appendix**). Furthermore, in Quang Truc young adults (18–25 years old) had the highest prevalence of asymptomatic malaria of 16.7% (19/114, RR = 1.37; OR = 1.44; 95% CI: 0.55 to 2.00) amongst all age groups, followed by 16.0% (8/50, RR = 1.24; OR = 1.29; 95% CI: 0.27 to 2.72 *P =* 0.37) amongst 56–65 years old and 13.8% (15/109, RR = 1.05; OR = 1.06; 95% CI: 0.37 to 1.47) in 26–35 years old (**S1 Table in S1 Appendix**). Because of the low number of positive cases for all age groups in the three communes, the 95% CI intervals were wide, with none of the ORs ratios reaching statistical significance (*P*<0.05) (**S1 Table in S1 Appendix**). Note that in low and moderate risk areas the highest prevalence of asymptomatic malaria was observed in the older age groups (**Fig 2, S1 Table in S1 Appendix**), in contrast to younger adults in the high risk area. These data might be useful for the identification of populations with the highest risk of asymptomatic malaria.

Most of the *Plasmodium* RT-qPCR positive samples had high Ct values, with mean (range) values of 34 (8–38), 33 (16–38) and 31 (7–38) for Dak Buk So, Dak Ngo and Quang Truc, respectively. The lowest Ct values were recorded for the three blood microscopy positive samples: DBS1026 (Ct = 8), QT528 (Ct = 7) and QT554 (Ct = 11) (**S2 Appendix.**). Based on the standards derived from the cultured laboratory *P*. *falciparum* D6 line, the RT-qPCR assay limit of quantification was 0.025 parasites/μL (Ct~32 cycles), after which the detection became stochastic. The participants’ mean parasite density in Quang Truc was the highest at approximately 0.050 parasites/μL or ~50 parasites/mL, whereas participants from Dak Ngo and Dak Buk So had lower mean parasite densities of less than 12 and 6 parasites/mL, respectively. The majority of the positive samples had parasite densities either equal to or below the limit of quantification: 90.9% (20/22), 77.4% (24/31) and 75.0% (54/72) in Dak Buk So, Dak Ngo and Quang Truc, respectively. Furthermore, *P*. *falciparum* parasites were detected by RT-qPCR in 32.0% (40/125) of positive cases: 27.4% (17/62) of participants from the 1^st^ cohort and 36.5% (23/63) from the 2^nd^ cohort. There were only three *P*. *vivax* infections detected amongst the positive cases by RT-qPCR, and all were from Quang Truc. For the remaining 82 malaria RT-qPCR-positive samples, the *Plasmodium* species could not be determined, presumably due to the very low parasite densities in the finger prick capillary blood samples.

### Adherence to malaria prevention measures and risk factors associated with asymptomatic malaria

Data on participants’ occupation, visiting forest, sleeping in the forest and LLINs and repellent use collected from the study participants were analysed to appreciate adherence to the malaria protective measures in the communities and to identify risk factors associated with asymptomatic malaria.

In Dak Buk So, Dak Ngo and Quang Truc 97.1%, 99.0% and 94.7% of participants, respectively, reported having LLINs or long lasting insecticide-treated hammocks at home in the commune or in the forest (**S3 Table in S1 Appendix**). However, of the study population, 66.0% (876/1,328), 57.3% (510/890) and 42.8% (253/591) reported using a bed net every night at home in Dak Buk So, Dak Ngo and Quang Truc, respectively. In these communes, 40.7% (540/1,328), 33.7% (300/890) and 29.3% (173/591) reported going and sleeping in the forest two weeks prior to enrolment in the study, however, of these participants only 30.6% (406/1,328), 8.3% (74/890) and 10.2% (60/591) reported sleeping under LLINs or hammocks in the forest (**S3 Table in S1 Appendix**), which encompasses 75.2%, 24.7% and 34.7% of LLINs owners in Dak Buk So, Dak Ngo and Quang Truc, respectively. Only 1.7% (10/591) of participants from Quang Truc and none in other communes acknowledged using a topical repellent.

Multiple logistic regression analysis did not reveal any significant associations of asymptomatic malaria prevalence with gender, body temperature, and pulse and respiratory function (data not shown). There was no significant difference (*P =* 0.41) in blood Hb concentration in participants with or without asymptomatic malaria, with mean concentrations of 11.9 g/dL (95% CI 11.3–12.3) and 12.0 g/dL (95% CI 12.1–12.4), respectively, nor for participants at each of the three communes (**S2 Table in S1 Appendix**). There were also no significant differences (*P*>0.05) in prevalence of individuals with lower blood Hb concentrations (<11 g/dL) for RT-qPCR-positive compared to RT-qPCR-negative participants from Dak Buk So, Dak Ngo and Quang Truc, as well as for combined data for all three communes (**S2 Table in S1 Appendix**).

Participants’ responses to the questionnaire were analysed to determine whether an associations exist between the presence or absence of asymptomatic malaria (RT-qPCR-positivity or negativity) and occupation, living in the forest and adherence to malaria protection measures using contingency table analyses (**S3 Table in S1 Appendix**). The majority of children (96.3%, 339/352) and adults (86.7%, 2,130/2,457) were students and farmers, respectively. Amongst the positive cases, 100% (9/9) of children were students and 87.1% (101/116) of adults were farmers. No significant associations between asymptomatic malaria and being a farmer or a student were found in the study population, whereas in Quang Truc the prevalence of asymptomatic malaria was significantly higher (*P* = 0.026) amongst forest rangers (OR = 3.8, 95% CI 1.41 to 10.50) (**S3 Table in S1 Appendix**).

Only in Dak Ngo there was a significant association between the number of participants reporting using bed nets rarely and asymptomatic malaria cases (OR 2.38, *P =* 0.048 95% CI 1.15 to 5.23) (**S3 Table in S1 Appendix**), whereas in Quang Truc asymptomatic malaria was linked to going to the forest (OR = 2.01, *P =* 0.008, 95% CI 1.19 to 3.28) and sleeping in the forest under a bed net OR = 2.48 (*P =* 0.011, 95% CI 1.30 to 4.80) (**S3 Table in S1 Appendix**).

## Discussion

As Vietnam heads towards malaria elimination it is important to develop strategies to deal with the asymptomatic malaria, a “silent reservoir” of malaria infections. Understanding the extent and the local context of the problem is a necessary step towards the goal of malaria elimination. This is the first study to report on the prevalence of asymptomatic malaria in the Central Highlands of Vietnam. The overall point prevalence of *Plasmodium* spp. in asymptomatic people was 1.7% (22/1,328) in Dak Buk So, 3.5% (31/890) in Dak Ngo and 12.2% (72/591) in Quang Truc. The RT-qPCR findings corroborate the malaria risk stratification by the NMCP of Dak Buk So, Dak Ngo, and Quang Truc being areas of low, moderate and high malaria risk areas, respectively. The prevalence of asymptomatic malaria in Quang Truc was similar to that reported in Binh Phuoc and Ninh Thuan provinces of Vietnam and western Cambodia, but lower than that in Lao-PDR [26]. By contrast, the prevalence of asymptomatic is significantly higher in high transmission areas of Papua New Guinea and Africa [13].

In this study, only children ≥10 to < 18 years of age were included, with 96.3% of children attending school across the three communes, whereas the majority of adults, (86.7%) were farmers, often working in or near forested areas. Absence of asymptomatic malaria in children in Dak Buk So and with only one child (1.1%, 1/93) of surveyed children with asymptomatic malaria in Dak Ngo suggests that children are at lower malaria risk in these areas. However, in Quang Truc 7.5% (8/107) of children had asymptomatic malaria compared to 13.2% (64/484) for adults. The higher prevalence of asymptomatic malaria in adults is likely to be due to different behavioural and occupational factors resulting in higher carriage of asymptomatic parasites in adults, particularly in the high risk area of Quang Truc.

Specific local factors appear to influence the prevalence of asymptomatic malaria, even in the same geographical region. In 2014, the prevalence of asymptomatic malaria in two villages in Myanmar along the China border was vastly different [34]. In the remote village of Mong Pawk, where the majority of the population were plantation workers, the prevalence of asymptomatic malaria was 9.9% (10/101) amongst children aged 5–17 years versus 7.8% (27/346) for all age groups, whereas in Laiza, a village harbouring refugees and migrants, the prevalence of asymptomatic malaria was much higher (26.6%, 37/139) in children (5–17 years old) compared to 16.0% (67/419) for all age groups. Note that Laiza can be considered a local malaria “hot spot”, where the incidence of symptomatic malaria infections was 8.1%, (34/419) [34] with all but one patient reporting a recent travel to an malaria endemic area, in contrast to no symptomatic malaria cases (0/346) reported in Mong Pawk [34]. The high incidence of symptomatic malaria in Laiza would more than likely contribute to the high prevalence of asymptomatic malaria infections. The wide range in the prevalence of asymptomatic malaria in both children and adults reported in our study further demonstrates the fluctuating multifactorial nature of asymptomatic malaria with local socio-demographical, geographical, and behavioural factors influencing the prevalence of asymptomatic malaria infections.

There were no significant differences in the prevalence of asymptomatic malaria cases between the two cohorts for each commune covering the end of the wet season and during the dry season, which was most likely due to only a 6-week interval between the two collection periods. Note that the data on symptomatic malaria cases provided by NMCP [33] **(S4 Table in S1 Appendix**) showed that there were no recorded malaria cases in Dak Buk So during the second half of 2018, yet 1.7% of the surveyed population had asymptomatic malaria parasites detected from the same commune. Interestingly, in February to March 2019 there were 6 symptomatic *P*. *falciparum* malaria cases detected in the low risk area of Dak Buk So **(S4 Table in S1 Appendix**). Of these cases, one was found to have asymptomatic malaria in our study prior to developing symptomatic malaria. In Dak Ngo and Quang Truc, the moderate and high risk areas, respectively, the prevalence of asymptomatic malaria cases also exceeded that of symptomatic malaria cases. Our data are consistent with previous observations that in low transmission areas the majority of malaria infections are submicroscopic and asymptomatic, but are responsible for 20–50% of the malaria transmission [16, 35–37]. Note that the data provided by NMCP may not capture all the malaria cases in the area as some patients from the communes may have been diagnosed with malaria and treated in another province. Additionally, patients may have self-medicated when they experienced malaria symptoms [38].

Although the present study was not designed to look for an association between asymptomatic and symptomatic malaria cases, the study findings confirm that there is a persistent dynamic reservoir of asymptomatic carriers that is not necessarily linked to or results in active malaria cases, but might play a role in sustaining malaria transmission in the area as observed in other studies in Vietnam and elsewhere [7, 13, 16, 36]. Active surveillance is required to identify asymptomatic malaria carriers to achieve elimination, especially in low risk areas, where the extent of asymptomatic malaria may not be fully apparent and appreciated.

With the exception of three microscopically positive yet asymptomatic malaria cases, the other 2,806 participants were microscopy negative, corroborating the findings that the remaining 122 RT-qPCR-positive malaria cases had very low parasite densities below the microscopy detection threshold, which under field conditions is considered to be about 100 parasites/μL [39, 40]. This is similar to the pyrogenic threshold that was estimated in areas, where the prevalence of asymptomatic malaria is low, such as in Senegal [17]. These findings are consistent with the very low parasite densities present in asymptomatic malaria infections in low transmission settings in contrast to higher parasitemias in moderate or high transmission areas, such as in Papua New Guinea [12, 30]. In addition, undetectable by RDT PfHRP2/3 antigens levels in asymptomatic submicroscopic falciparum infections in this study are suggestive of very low parasite densities or sub-patent infections but would rule out very recent acute malaria infections, which is consistent with the fact that none of the participants sought antimalarial treatment during the 28 days prior to enrolment. Recent data from whole genome sequencing of 180 *P*. *falciparum* isolates from Vietnam demonstrated that the frequency of *hrp2/hrp3* deletions was very low in Vietnam, with less than 1.1% and 3.3% for *hrp2* and *hrp3* genes, respectively [41], and therefore, unlikely to significantly affect the sensitivity of detection by RDT.

A distinct advantage of using the RNA-based 18S SSU rRNA RT-qPCR assay is its greater sensitivity compared to DNA-based standard qPCR in detecting submicroscopic parasitemia, with an LOD of 0.02–0.002 parasite/μL of blood [30, 42, 43]. However, the downstream assay used in this study for *P*. *vivax* speciation was at least 10–100 times less sensitive due to several reasons outlined in the review by Gruenberg *et al*. [44]. The estimated mean parasite densities in the asymptomatic malaria cases from the three communes ranged between 6 and 50 parasites/mL, with the majority of positive samples having parasite densities close to the limit of quantification. This may explain why it was not possible to detect more *P*. *vivax* cases amongst RT-qPCR positive samples to accurately determine the prevalence of *P*. *vivax* asymptomatic infections in the three communes. Because of this limitation, only three asymptomatic *P*. *vivax* malaria cases, all detected in Quang Truc, were identified in this study compared to 32.0% (40/125) asymptomatic *P*. *falciparum* cases. Note that according to NMCP data in 2018, the proportion of symptomatic *P*. *vivax* infections was 42.9% (6/14) for Dak Ngo and 47.4% (27/57) for Quang Truc (**Table 2**) in subjects presenting to the communal health stations and district hospital. However, the distribution of species in asymptomatic malaria may not resemble that of symptomatic cases, with *P*. *vivax* having a greater propensity (64% to 100% of infections) to remain asymptomatic compared with *P*. *falciparum* [3, 45, 46].

In contrast to symptomatic malaria, there was no associations between asymptomatic malaria and participants’ characteristics, such as body temperature, pulse,respiratory rate and blood Hb concentrations. In other studies anemia has been linked not only to symptomatic malaria with high parasitemia, but is also associated with asymptomatic or “chronic” malaria [22, 47]. The very low parasite densities in the study population may explain the observation that there was no statistically significant difference in the blood Hb concentrations observed between the RT-qPCR-positive and -negative participants. It is quite possible that other health conditions such as concomitant hemoglobinopathies, nutritional deficiencies, and intestinal helminth infections may also mask anemia due to asymptomatic malaria [47].

The majority of the 125 RT-qPCR positive asymptomatic participants were adult farmers living or working in or near forested areas of the communes and thus were most likely to be exposed to malaria, since malaria transmission mainly occurs in remote forested areas of the Central Highlands of Vietnam and along the international borders with Cambodia and Lao-PDR [23, 48]. In the high malaria risk area of Quang Truc, significant associations were found between asymptomatic malaria prevalence and the following: participants’ occupation a forest ranger (OR = 3.80, *P* = 0.026) or going to the forest two weeks prior to enrolment (OR = 2.01, *P =* 0.008) and sleeping in the forest under a bed net (OR = 2.48, *P =* 0.011). These findings further support the notion that forest goers are at the highest risk to malaria exposure and interventions need to be prioritized for this group, consistent with previous recommendations [49]. As no significant association risk factors could be identified in the low and moderate malaria risk areas, malaria exposure in those areas may be more heterogenic and focal, similar to findings in the neighbouring Phu Yen province in Central Vietnam [38, 50].

In Central Vietnam malaria transmission is sustained by a variety of *Anopheles* mosquito vectors in forested areas and on forest fringes, with primary malaria vectors *Anopheles dirus* and *An*. *minimus* predominantly biting outdoors early at dusk, as well as secondary vectors that bite from dusk to down [51, 52]. LLINs and long-lasting insecticide-treated hammocks are still considered major malaria prevention measures, recommended by NMCP and distributed amongst exposed populations by the Global Fund to Fight AIDS, Tuberculosis, and Malaria [49]. Importantly, to maintain their efficacy, LLINs and hammocks require regular re-treatment or replacement. Despite the rationale to use topical repellents for protection against mosquitoes during early evening and morning hours, it has neither been widely accepted by the population nor its use demonstrated to significantly reduce the prevalence of malaria infections, when used in addition to LLINs in clinical trials [53]. In the present study, LLINs and hammocks coverage was very high at 95% to 99%, however, only 42.8% to 66.0% of participants reported using them every night and 41.3% to 59.9% confirmed using treated bed nets.

The highest adherence to protective measures was in the low risk area (Dak Buk So), whereas in the high risk area (Quang Truc) the lowest usage of LLINs and hammocks was reported (**S3 Table in S1 Appendix**). In the moderate risk area (Dak Ngo), infrequent sleeping under LLINs was significantly associated with asymptomatic malaria prevalence. In Quang Truc the asymptomatic malaria prevalence was associated with going to the forest and sleeping in the forest under a bed net (**S3 Table in S1 Appendix**), likely indicating that spending a significant amount of time and sleeping in the forest is associated with the risk of having asymptomatic malaria. The relatively low utilization of LLINs and regular retreatment or replacement of LLINs in all three communes needs to be addressed in order to further reduce both, symptomatic and asymptomatic malaria in the area. Furthermore, our results confirmed that only 1.7% of participants used a topical repellent in Quang Truc, with none reported by participants in the other two communes, which was also observed in other areas of Central Vietnam and GMS [38, 53].

This study demonstrates that asymptomatic malaria is present in low, moderate and high risk areas in Dak Nong province in the Central Highlands of Vietnam, with prevalence greatly exceeding that of symptomatic malaria at each study site. Findings from this and other cross-sectional surveys of asymptomatic malaria [7, 23, 25, 26] confirm the common occurrence of sub-patent malaria infections in low transmission settings in Vietnam. However, identifications of risk factors and predictors for asymptomatic malaria remain challenging and its detection still requires costly laboratory methods. In order to achieve malaria elimination in Vietnam, further studies are required to design and evaluate targeted interventions against asymptomatic malaria.

## Conclusion

This is the first study to report on the point prevalence of asymptomatic malaria in the Central Highlands of Vietnam, with a prevalence ranging from 1.7% to 12.2% for participants residing in three communes in Tuy Duc district, Dak Nong province. Of the several risk factors examined, going to the forest two weeks prior to enrolment into the study and sleeping in the forest, as well as rarely sleeping under bed net at home in the commune were significantly associated with asymptomatic malaria in different malaria risk areas. To achieve malaria elimination, intervention programs must target asymptomatic malaria carriers to prevent transmission of persistent malaria infections in low transmission areas.

## Supporting information

S1 Appendix (PDF)

S2 Appendix RT-qPCR results. (XLSX)

S3 Appendix (XLSX)
