# Supplementary material for: Cross-sectional survey of asymptomatic malaria in Dak Nong province in the Central Highlands of Vietnam for the malaria elimination roadmap
Source: PLoS One. 2021 Oct 20;16(10):e0258580. doi: 10.1371/journal.pone.0258580 (PMC8528296; doi:10.1371/journal.pone.0258580)
Supplement: S1 Appendix — (PDF) [file pone.0258580.s001.pdf]

Appendix 1.

Supporting information

Cross-Sectional Survey of Asymptomatic Malaria in Dak Nong Province in the Central Highlands of Vietnam for the Malaria Elimination Roadmap

Huynh Hong Quang,[1§] Marina Chavchich,[2§] Nguyen Thi Minh Trinh,[1] Nguyen Duc Manh,[3] Michael D. Edstein,[2] Nicholas J. Martin[4] and Kimberly A. Edgel[4]

[1] Institute of Malariology, Parasitology and Entomology Quy Nhon, Vietnam,

[2] Australian Defence Force Malaria and Infectious Disease Institute, Brisbane, Australia

[3] Military Institute of Preventive Medicine, Hanoi, Vietnam,

[4] U.S. Naval Medical Research Unit TWO, Singapore

§ These authors contributed equally to this work.

S1. Table. Age distribution of asymptomatic malaria in the three communes (A), in Dak Buc So (B), Dak Ngo (C) and Quang Truc (D) in Tuy Duc district, Dak Nong province, Central Highlands of Vietnam in 2018-2019**.**

**A.**	**All three communes**							
**Age (years)**	**Total No. in three communes**	**No. RT-qPCR (+)**	**No. RT-qPCR (-)**	**Prevalence (%)**	**Relative Risk**	**Odds Ratio**	**95% CI, Lower and Upper limits**	
10-15	333	8	325	2.4	0.51	0.50	0.24	1.01
16-17	19	1	18	5.3	1.15	1.12	0.11	6.35
**Children**	**352**	**9**	**343**	**2.6**	**0.54**	**0.53**	**0.28**	**1.02**
18-25	550	30	520	5.5	1.21	1.22	0.80	1.85
26-35	713	25	688	3.5	0.67	0.66	0.42	1.04
36-45	445	20	425	4.5	0.94	0.94	0.58	1.54
46-55	338	16	322	4.7	1.00	1.00	0.57	1.72
56-65	260	16	244	6.2	1.35	1.38	0.78	2.32
66-75	107	8	99	7.5	1.68	1.68	0.80	3.45
76-85	37	1	36	2.7	1.63	0.56	0.05	3.17
>85	7	0	7	0.0	0.00	0.00	0.00	11.15
**Adults**	**2457**	**116**	**2341**	**4.7**	**1.00**			
**Total**	**2809**	**125**	**2684**	**4.4**	**1.00**			
**B.**	**Dak Buk So**							
**Age (years)**	**No. in commune**	**No. RT-qPCR (+)**	**No. RT-qPCR (-)**	**Prevalence (%)**	**Relative Risk**	**Odds Ratio**	**95% CI, Lower and Upper limits**	
10-15	144	0	144	0.0	0.00	0.00	0.00	1.26
16-17	8	0	8	0.0	0.00	0.00	0.00	25.93
**Children**	**152**	**0**	**152**	**0.0**	**0.00**	**0.00**	**0.00**	**1.19**
18-25	225	4	221	1.8	0.94	0.94	0.34	2.71
26-35	352	4	348	1.1	0.52	0.51	0.19	1.48
36-45	221	3	218	1.4	0.68	0.68	0.21	2.25
46-55	158	4	154	2.5	1.43	1.44	0.52	4.19
56-65	140	4	136	2.9	1.64	1.66	0.60	4.85
66-75	55	3	52	5.5	3.22	3.35	1.02	10.62
76-85	21	0	21	0.0	0.00	0.00	0.00	9.51
>85	4	0	4	0.0	0.00	0.00	0.00	56.30
**Adults**	**1176**	**22**	**1154**	**1.9**				
**Total**	**1328**	**22**	**1306**	**1.7**	**1.00**			

C.	Dak Ngo							
**Age (years)**	**No. in commune**	**No. RT-qPCR (+)**	**No. RT-qPCR (-)**	**Prevalence (%)**	**Relative Risk**	**Odds Ratio**	**95% CI, Lower and Upper limits**	
10-15	91	1	90	1.1	0.29	0.28	0.03	1.67
16-17	2	0	2	0.0	0.00	0.00	0.00	56.02
**Children**	**93**	**1**	**92**	**1.1**	**0.29**	**0.28**	**0.03**	**1.64**
18-25	211	7	204	3.3	0.85	0.84	0.34	1.90
26-35	252	6	246	2.4	0.54	0.53	0.23	1.26
36-45	121	6	115	5.0	1.40	1.42	0.60	3.43
46-55	104	4	100	3.8	1.03	1.03	0.38	2.76
56-65	70	4	66	5.7	1.60	1.63	0.60	4.49
66-75	27	2	25	7.4	2.04	2.12	0.47	8.67
76-85	9	1	8	11.1	3.02	3.27	0.29	22.89
>85	3	0	3	0.0	0.00	0.00	0.00	30.00
**Adults**	**797**	**30**	**767**	**3.8**	**1.00**			
**Total**	**890**	**31**	**859**	**3.5**	**1.00**			
`								

D.	Quang Truc							
**Age (years)**	**No. in commune**	**No. RT-qPCR (+)**	**No. RT-qPCR (-)**	**Prevalence (%)**	**Relative Risk**	**Odds Ratio**	**95% CI, Lower and Upper limits**	
10-15	98	7	91	7.1	0.54	0.50	0.07	5.47
16-17	9	1	8	11.1	0.84	0.82	0.25	1.10
**Children**	**107**	**8**	**99**	**7.5**	**0.57**	**0.53**	**0.80**	**2.57**
18-25	114	19	95	16.7	1.37	1.44	0.55	2.00
26-35	109	15	94	13.8	1.05	1.06	0.37	1.47
36-45	103	11	92	10.7	0.77	0.74	0.35	1.61
46-55	76	8	68	10.5	0.77	0.74	0.60	2.79
56-65	50	8	42	16.0	1.24	1.29	0.27	2.72
66-75	25	3	22	12.0	0.90	0.89	0.00	3.65
76-85	7	0	7	0.0	0.00	0.00		
>85		0	0	0.0	0.00	0.00	0.91	3.96
**Adults**	**484**	**64**	**420**	**13.2**	**1.00**			
**Total**	**591**	**72**	**519**	**12.2**	**1.00**			

S2 Table. Blood haemoglobin (Hb) mean concentration (A) and prevalence of mild (Hb 8 to 11 g/dL) and moderate (Hb <8 g/dL) anemia (B) in RT-qPCR-positive and RT-qPCR-negative participants in Dak Buk So, Dak Ngo and Quang Truc communes, Tuy Duc district, Dak Nong province, Central Highlands of Vietnam.

A.

	**Dak Buk So**					**Dak Ngo**					**Quang Truc**					**All Communes**				
	**RT-qPCR-positive**		**RT-qPCR- negative**		***P-value***	**RT-qPCR-positive**		**RT-qPCR- negative**		***P-value***	**RT-qPCR-positive**		**RT-qPCR- negative**		***P-value***	**RT-qPCR-positive**		**RT-qPCR- negative**		***P-value***
	**Mean**	**95% CI**	**Mean**	**95% CI**		**Mean**	**95% CI**	**Mean**	**95% CI**		**Mean**	**95% CI**	**Mean**	**95% CI**		**Mean**	**95% CI**	**Mean**	**95% CI**	
**Hb (g/dL)**	12.68	11.76-13.60	12.06	11.96-12.17	*0.13*	11.48	10.81-12.15	11.73	11.62-11.83	*0.39*	11.75	11.25-12.25	12.23	12.05-12.42	*0.08*	11.85	11.25-12.25	11.99	12.05-12.42	*0.41*

B.

	**Dak Buk So**				**Relative Risk/*P-value***	**Dak Ngo**				**Relative Risk/*P-value***	**Quang Truc**				**Relative Risk/*P-value***	**All Communes**				**Relative Risk/*P-value***
**Hb (g/dL)**	**RT-qPCR-positive**		**RT-qPCR- negative**			**RT-qPCR-positive**		**RT-qPCR- negative**			**RT-qPCR-positive**		**RT-qPCR- negative**			**RT-qPCR-positive**		**RT-qPCR- negative**		
	**n**	**%**	**n**	**%**		**n**	**%**	**n**	**%**		**n**	**%**	**n**	**%**		**n**	**%**	**n**	**%**	
**<8**	1	4.5%	22	1.7%		1	3.2%	8	0.9%		2	3%	13	3%		4	**3%**	43	**2%**	
**8 to 11**	4	18%	296	23%		9	29%	242	28%		21	29%	114	22%		34	**27%**	652	**24%**	
**≥11**	17	77%	978	75%	0.93	21	68%	618	71%	1.12	49	68%	392	76%	1.31	87	**70%**	1988	**74%**	1.17
**Total**	**22**	**100%**	**1296**	**100%**	***>0.99***	**31**	**100%**	**868**	**100%**	***0.69***	**72**	**100%**	**519**	**100%**	***0.19***	**125**	**100%**	**2683**		***0.30***
**Total**	**1318**					**899**					**591**					**2808**				

S3 Table. Adherence to malaria protection measures and risk factors for asymptomatic malaria in Dak Buk So, Dak Ngo and Quang Truc Tuy Duc district, Dak Nong province, Central Highlands of Vietnam in 2018-2019.

Factors Analysed	% of Total Study population, n/N	Data analyzed	Number of "Exposed"	Number of "Not exposed"	Total Number	% in RT-qPCR (+) or RT-qPCR (-) of all "Exposed" to risk	% in RT-qPCR (+) or RT-qPCR of all "Not exposed" to risk	"Exposed" Percentage of grand total	"Not exposed" Percentage of grand total	"Exposed" (% in RT-qPCR (+) or RT-qPCR (-))	"Not exposed" (% in RT-qPCR (+) or RT-qPCR (-))	Relative Risk, 95% CI	Odds ratio, 95% CI	*P-value*
**Dak Buk So**														
Farmers (Y/N)	70.9%	RT-qPCR (+)	15	7	22	1.6%	1.8%	1.1%	0.5%	68.2%	31.8%	0.88	0.88	**0.814**
	941/1328	RT-qPCR (-)	926	380	1306	98.4%	98.2%	69.7%	28.6%	70.9%	29.1%	0.37 to 2.09	0.37 to 2.33	
**Total**			**941**	**387**	**1328**	**100%**	**100%**	**70.9%**	**29.1%**					
Students (Y/N)	14.4%	RT-qPCR (+)	0	22	22	0%	1.9%	0.0%	1.7%	0.0%	100%	0	0	**0.061**
	191/1328	RT-qPCR (-)	191	1115	1306	100.0%	98.1%	14.4%	84.0%	14.6%	85.4%	0.00 to 1.02	0.000 to 0.91	
**Total**			**191**	**1137**	**1328**	**100%**	**100%**	**14.4%**	**85.6%**					
Slept in forest 2 weeks prior (Y/N)	40.7%	RT-qPCR (+)	11	11	22	2.04%	1.40%	0.8%	0.8%	50.0%	50.0%	1.46	1.47	**0.388**
	540/1328	RT-qPCR (-)	529	777	1306	98.0%	98.6%	39.8%	58.5%	40.5%	59.5%	0.65 to 3.27	0.66 to 3.25	
**Total**			**540**	**788**	**1328**	**100%**	**100%**	**40.7%**	**59.3%**					
Slept in forest weeks prior under bed net (Y/N)	30.6%	RT-qPCR (+)	9	13	22	2.2%	1.41%	0.7%	1.0%	40.9%	59.1%	1.57	1.59	**0.350**
	406/1328	RT-qPCR (-)	397	909	1306	97.8%	1	29.9%	68.5%	30.4%	69.6%	0.69 to 3.56	0.64 to 3.72	
**Total**			**406**	**922**	**1328**	**100%**	**100%**	**30.6%**	**69.4%**					
Lived in the forest - slept few times a week under bednet (Y/N)	12.0%	RT-qPCR (+)	3	19	22	1.9%	1.6%	0.2%	1.4%	13.6%	86.4%	1.16	1.16	**0.740**
	159/1328	RT-qPCR (-)	156	1150	1306	98.1%	98.4%	11.8%	86.6%	11.9%	88.1%	0.37 to 3.60	0.36 to 3.52	
**Total**			**159**	**1169**	**1328**	**100%**	**100%**	**12.0%**	**88.0%**					
Bed net at home or hammock in the forest (Y/N)	97.1%	RT-qPCR (+)	20	2	22	1.6%	5.3%	1.5%	0.2%	90.9%	9.1%	0.29	0.28	**0.129**
	1290/1328	RT-qPCR (-)	1270	36	1306	98.5%	94.7%	95.6%	2.7%	97.2%	2.8%	0.08 to 1.13	0.07 to 1.27	
**Total**			**1290**	**38**	**1328**	**100%**	**100%**	**97.1%**	**2.9%**					
Bednet at home - Every night (Y/N)	66.0%	RT-qPCR (+)	17	5	22	1.9%	1.1%	1.3%	0.4%	77.3%	22.7%	1.75	1.77	**0.365**
	876/1328	RT-qPCR (-)	859	447	1306	98.1%	98.9%	64.7%	33.7%	65.8%	34.2%	0.68 to 4.56	0.68 to 4.41	
**Total**			**876**	**452**	**1328**	**100%**	**100%**	**66.0%**	**34.0%**					
Bednet at home - Rarely (Y/N)	10.2%	RT-qPCR (+)	0	22	22	0.0%	1.8%	0.0%	1.7%	0.0%	100%	0	0	**0.157**
	135/1328	RT-qPCR (-)	135	1171	1306	100%	98.2%	10.2%	88.2%	10.3%	89.7%	0.00 to 1.51	0.00 to 1.37	
**Total**			**135**	**1193**	**1328**	**100%**	**100%**	**10.2%**	**89.8%**					
Bed net is treated (Y/N)	59.9%	RT-qPCR (+)	13	9	22	1.6%	1.7%	1.0%	0.7%	59.1%	40.9%	0.97	0.97	**>0.999**
	795/1328	RT-qPCR (-)	782	524	1306	98.4%	98.3%	58.9%	39.5%	59.9%	40.1%	0.43 to 2.20	0.41 to 2.38	
**Total**			**795**	**533**	**1328**	**100%**	**100%**	**59.9%**	**40.1%**					
Repellent (Y/N)	0%	RT-qPCR (+)	0	22	22	0.0%	1.7%	0.0%	1.7%	0.0%	100%	0	0	**>0.999**
	0/1328	RT-qPCR (-)	4	1302	1306	100%	98.3%	0.3%	98.0%	0.3%	99.7%	0.00 to 31.77	0 to 63.73	
**Total**			**4**	**1324**	**1328**	**100%**	**100%**	**0.3%**	**99.7%**					

Factors Analysed	% of Total Study population, n/N	Data analyzed	Number of "Exposed"	Number of "Not exposed"	Total Number	% in RT-qPCR (+) or RT-qPCR (-) of all "Exposed" to risk	% in RT-qPCR (+) or RT-qPCR of all "Not exposed" to risk	"Exposed" Percentage of grand total	"Not exposed" Percentage of grand total	"Exposed" (% in RT-qPCR (+) or RT-qPCR (-))	"Not exposed" (% in RT-qPCR (+) or RT-qPCR (-))	Relative Risk/95% CI	Odds ratio/95% CI	*P-value*
**Dak Ngo**														
Farmers (Y/N)	86.5%	RT-qPCR (+)	30	1	31	3.9%	0.8%	3.4%	0.1%	96.8%	3.2%	4.68	4.82	**0.108**
	770/890	RT-qPCR (-)	740	119	859	96.1%	99.2%	83.2%	13.4%	86.2%	13.9%	0.83 to 27.05	0.83 to 49.99	
**Total**			**770**	**120**	**890**	**100%**	**100%**	**86.5%**	**13.5%**					
Forest goers (Y/N)	3.5%	RT-qPCR (+)	0	31	5	0%	0.56%	0.0%	3.5%	0.0%	100.0%	0	0	**>0.999**
	5/890	RT-qPCR (-)	5	854	885	100%	99.4%	0.6%	96.0%	0.6%	99.42%	0.0 to 13.01	0.000 to 19.43	
**Total**		31	**5**	**885**	**890**	**100%**	**100%**	**0.56%**	**99.4%**					
Students (Y/N)	10.4%	RT-qPCR (+)	1	30	31	1.1%	3.8%	0.1%	3.4%	3.2%	96.8%	0.29	0.28	**0.241**
	93/890	RT-qPCR (-)	92	767	859	98.9%	96.2%	10.3%	86.2%	10.7%	89.3%	0.049 to 1.60	0.03 to 1.64	
**Total**			**93**	**797**	**890**	**100%**	**100%**	**10.5%**	**89.6%**					
Slept in forest 2 weeks prior (Y/N)	33.7%	RT-qPCR (+)	11	20	31	3.7%	3.4%	1.2%	2.3%	35.5%	64.5%	1.08	1.09	**0.848**
	300/890	RT-qPCR (-)	289	570	859	96.3%	96.6%	32.5%	64.0%	33.6%	66.4%	0.53 to 2.19	0.50 to 2.31	
**Total**			**300**	**590**	**890**	**100%**	**100%**	**33.7%**	**66.3%**					
Slept in forrest weeks prior under bed net (Y/N)	8.3%	RT-qPCR (+)	3	28	31	4.1%	3.4%	0.3%	3.2%	9.7%	90.3%	1.18	1.19	**0.738**
	74/890	RT-qPCR (-)	71	788	859	96.0%	96.6%	8.0%	88.5%	8.3%	91.7%	0.39 to 3.48	0.37 to 3.75	
**Total**			**74**	**816**	**890**	**100%**	**100%**	**8.3%**	**91.7%**					
Lived in the forest - slept few times a week under bednet (Y/N)	24.6%	RT-qPCR (+)	7	24	31	3.2%	3.6%	0.8%	2.7%	22.6%	77.4%	0.89	0.89	**>0.999**
	219/890	RT-qPCR (-)	212	647	859	96.8%	96.4%	23.8%	72.7%	24.7%	75.3%	0.40 to 1.99	0.36 to 1.98	
**Total**			**219**	**671**	**890**	**100%**	**100%**	**24.6%**	**75.4%**					
Bed net at home or hammock in the forest (Y/N)	99.0%	RT-qPCR (+)	31	0	31	3.5%	0.0%	3.5%	0.0%	100.0%	0.0%	Infinity	Infinity	**>0.999**
	881/890	RT-qPCR (-)	850	9	859	96.5%	100.0%	95.5%	1.0%	99.0%	1.1%	0.11 to Infinity	0.09 to Infinity	
**Total**			**881**	**9**	**890**	**100%**	**100%**	**99.0%**	**1.0%**					
Bednet at home - Every night (Y/N)	57.3%	RT-qPCR (+)	15	16	31	2.9%	4.2%	1.7%	1.8%	48.4%	51.6%	0.70	0.69	**0.357**
	510/890	RT-qPCR (-)	495	364	859	97.1%	95.8%	55.6%	40.9%	57.6%	42.4%	0.35 to 1.38	0.34 to 1.37	
**Total**			**510**	**380**	**890**	**100%**	**100%**	**57.3%**	**42.7%**					
Bednet at home - Rarely (Y/N)	17.2%	RT-qPCR (+)	10	21	31	6.5%	2.9%	1.1%	2.4%	32.3%	67.7%	2.29	2.38	**0.048***
	153/890	RT-qPCR (-)	143	716	859	93.5%	97.2%	16.1%	80.5%	16.7%	83.4%	1.11 to 4.67	1.15 to 5.23	
**Total**			**153**	**737**	**890**	**100%**	**100%**	**17.2%**	**82.8%**					
Bed net is treated (Y/N)	47.1%	RT-qPCR (+)	11	20	31	2.6%	4.3%	1.2%	2.3%	35.5%	64.5%	0.62	0.61	**0.205**
	419/890	RT-qPCR (-)	408	451	859	97.4%	95.8%	45.8%	50.7%	47.5%	52.5%	0.30 to 1.25	0.28 to 1.29	
**Total**			**419**	**471**	**890**	**100%**	**100%**	**47.1%**	**52.9%**					
Repellent (Y/N)	0%	RT-qPCR (+)	0	31	31		3.5%	0.0%	3.5%	0.0%	100%	0		**>0.999**
	0/890	RT-qPCR (-)	0	859	859		96.5%	0.0%	96.5%	0.0%	100%	0.0 to 1.0		
**Total**			**0**	**890**	**890**	**0.0%**	**100%**	**0.0%**	**100%**					

Factors Analysed	% of Total Study population, n/N	Data analyzed	Number of "Exposed"	Number of "Not exposed"	Total Number	% in RT-qPCR (+) or RT-qPCR (-) of all "Exposed" to risk	% in RT-qPCR (+) or RT-qPCR of all "Not exposed" to risk	"Exposed" Percentage of grand total	"Not exposed" Percentage of grand total	"Exposed" (% in RT-qPCR (+) or RT-qPCR (-))	"Not exposed" (% in RT-qPCR (+) or RT-qPCR (-))	Relative Risk/95% CI	Odds ratio/95% CI	*P-value*
**Quang Truc**														
Farmers (Y/N)	74.3%	RT-qPCR (+)	56	16	72	12.8%	10.5%	9.5%	2.7%	77.8%	22.2%	1.21	1.24	**0.565**
	439/591	RT-qPCR (-)	383	136	519	87.2%	89.5%	64.8%	23.0%	73.8%	26.2%	0.73 to 2.05	0.71 to 2.21	
**Total**			**439**	**152**	**591**	**100%**	**100%**	**74.3%**	**25.7%**					
Forest rangers (Y/N)	2.5%	RT-qPCR (+)	5	67	72	33%	12.2%	0.8%	11.3%	6.9%	93.1%	2.87	3.80	**0.026***
	15/591	RT-qPCR (-)	10	509	519	67%	87.8%	1.7%	86.1%	1.9%	98.1%	1.27 to 5.31	1.41 to 10.50	
**Total**			**15**	**576**	**591**	**100%**	**100%**	**2.5%**	**97.5%**					
Students (Y/N)	17.9%	RT-qPCR (+)	9	63	72	8.5%	13.0%	1.5%	10.7%	12.5%	87.5%	0.65	0.62	**0.251**
	106/591	RT-qPCR (-)	97	422	519	91.5%	87.0%	16.4%	71.4%	18.7%	81.3%	0.34 to 1.24	0.30 to 1.29	
**Total**			**106**	**485**	**591**	**100%**	**100%**	**17.9%**	**82.1%**					
Slept in forest 2 weeks prior (Y/N)	29.3%	RT-qPCR (+)	31	41	72	17.9%	9.8%	5.3%	6.9%	43.1%	56.9%	1.83	2.01	**0.008***
	173/591	RT-qPCR (-)	142	377	519	82.1%	90.2%	24.0%	63.8%	27.4%	72.6%	1.19 to 2.79	1.19 to 3.28	
**Total**			**173**	**418**	**591**	**100%**	**100%**	**29.3%**	**70.7%**					
Slept in forest weeks prior under bed net (Y/N)	10.2%	RT-qPCR (+)	14	58	72	23.3%	10.9%	2.4%	9.8%	19.4%	80.6%	2.14	2.48	**0.011***
	60/591	RT-qPCR (-)	46	473	519	76.7%	89.1%	7.8%	80.0%	8.9%	91.1%	1.25 to 3.48	1.30 to 4.80	
**Total**			**60**	**531**	**591**	**100%**	**100%**	**10.2%**	**89.8%**					
Lived in the forest - slept few times a week under bednet (Y/N)	17.4%	RT-qPCR (+)	17	55	72	16.5%	11.3%	2.9%	9.3%	23.6%	76.4%	1.46	1.56	**0.139**
	103/591	RT-qPCR (-)	86	433	519	83.5%	88.7%	14.6%	73.3%	16.6%	83.4%	0.88 to 2.37	0.88 to 2.77	
**Total**			**103**	**488**	**591**	**100%**	**100%**	**17.4%**	**82.6%**					
Bed net at home or hammock in the forest (Y/N)	94.9%	RT-qPCR (+)	69	3	72	12.3%	10.0%	11.7%	0.5%	95.8%	4.2%	1.23	1.26	**>0.999**
	561/591	RT-qPCR (-)	492	27	519	87.7%	90.0%	83.3%	4.6%	94.8%	5.2%	0.47 to 3.62	0.40 to 4.05	
**Total**			**561**	**30**	**591**	**100%**	**100%**	**94.9%**	**5.1%**					
Bednet at home - Every night (Y/N)	42.8%	RT-qPCR (+)	34	38	72	13.4%	11.2%	5.8%	6.4%	47.2%	52.8%	1.20	1.23	**0.447**
	253/591	RT-qPCR (-)	219	300	519	86.6%	88.8%	37.1%	50.8%	42.2%	57.8%	0.78 to 1.84	0.75 to 1.98	
**Total**			**253**	**338**	**591**	**100%**	**100%**	**42.8%**	**57.2%**					
Bednet at home - Rarely (Y/N)	25.9%	RT-qPCR (+)	17	55	72	11.1%	12.6%	2.9%	9.3%	23.6%	76.4%	0.88	0.87	**0.774**
	153/591	RT-qPCR (-)	136	383	519	88.9%	87.4%	23.0%	64.8%	26.2%	73.8%	0.53 to 1.46	0.49 to 1.56	
**Total**			**153**	**438**	**591**	**100%**	**100%**	**25.9%**	**74.1%**					
Bed net is treated (Y/N)	41.3%	RT-qPCR (+)	35	37	72	14.3%	10.7%	5.9%	6.3%	48.6%	51.4%	1.35	1.40	**0.202**
	244/591	RT-qPCR (-)	209	310	519	85.7%	89.3%	35.4%	52.5%	40.3%	59.7%	0.88 to 2.06	0.86 to 2.27	
**Total**			**244**	**347**	**591**	**100%**	**100%**	**41.3%**	**58.7%**					
Repellent (Y/N)	1.7%	RT-qPCR (+)	1	71	72	10.0%	12.2%	0.2%	12.0%	1.4%	98.6%	0.82	0.80	**>0.999**
	10/591	RT-qPCR (-)	9	510	519	90.0%	87.8%	1.5%	86.3%	1.7%	98.3%	0.15 to 3.40	0.07 to 4.96	
**Total**			**10**	**581**	**591**	**100%**	**100%**	**1.7%**	**98.3%**					

S4. Table. Malaria incidence confirmed by blood film microscopy in Dak Buk So, Dak Ngo and Quang Truc communes, Tuy Duc district, Dak Nong province, Central Highlands of Vietnam during 2018-2019*.

Communes	Malaria cases/species	2018													2019												
		Jan	Feb	Mar	Apr	May	Jun	Jul	Aug	Sep	Oct	Nov	Dec	Total 2018	Jan	Feb	Mar	Apr	May	Jun	Jul	Aug	Sep	Oct	Nov	Dec	Total 2019
Dak Buk So	*P. falciparum*				1									**1**		3	3										**6**
	P. vivax													**0**	1				1								**2**
	Mixed species																										**0**
	**Total**				**1**									**1**	**1**	**3**	**3**		**1**								**8**
Dak Ngo	*P. falciparum*	5		2		1								**8**	1	1								1			**3**
	P. vivax		1			1		1		1		2		**6**						1				1			**2**
	Mixed species																										**0**
	**Total**	**5**	**1**	**2**		**2**		**1**		**1**		**2**		**14**	**1**	**1**				**1**				**2**			**5**
Quang Truc	*P. falciparum*		6	10	2	1	1			1	1	1	5	**28**	8	1	3	4	3	2	1	1	1		1		**25**
	P. vivax	3	6	10	3		3			1		1	1	**28**	10	4	4	2	3	2				1	4	5	**35**
	Mixed species			1										**1**													**0**
	**Total**	**3**	**12**	**21**	**5**	**1**	**4**			**2**	**1**	**2**	**6**	**57**	**18**	**5**	**7**	**6**	**6**	**4**	**1**	**1**	**1**	**1**	**5**	**5**	**60**

*Mid-wet season is from August to October. Dry season is from December to February. Data provided by the National Malaria Control Program in Tuy Duc district, Dak Nong province.
